# Cytochrome P450 2E1 Gene Polymorphisms/Haplotypes and Anti-Tuberculosis Drug-Induced Hepatitis in a Chinese Cohort

**DOI:** 10.1371/journal.pone.0057526

**Published:** 2013-02-27

**Authors:** Shaowen Tang, Xiaozhen Lv, Yuan Zhang, Shanshan Wu, Zhirong Yang, Yinyin Xia, Dehua Tu, Peiyuan Deng, Yu Ma, Dafang Chen, Siyan Zhan

**Affiliations:** 1 Department of Epidemiology and Biostatistics, School of Public Health, Peking University Health Science Centre, Beijing, China; 2 Department of Epidemiology and Biostatistics, School of Public Health, Nanjing Medical University, Nanjing, China; 3 Center for Tuberculosis Control and Prevention, Chinese Center for Disease Control and Prevention, Beijing, China; 4 Department of Tuberculosis Treatment, Beijing Institute for Tuberculosis Control, Beijing, China; 5 Center for Drug Reassessment, State Food and Drug Administration, Beijing, China; 6 Department of Tuberculosis Treatment, Beijing Tuberculosis and Thoracic Tumor Research Institute, Beijing, China; Vanderbilt University Medical Center, United States of America

## Abstract

**Objective:**

The pathogenic mechanism of anti-tuberculosis (anti-TB) drug-induced hepatitis is associated with drug metabolizing enzymes. No tagging single-nucleotide polymorphisms (tSNPs) of cytochrome P450 2E1(CYP2E1) in the risk of anti-TB drug-induced hepatitis have been reported. The present study was aimed at exploring the role of tSNPs in *CYP2E1* gene in a population-based anti-TB treatment cohort.

**Methods and Design:**

A nested case-control study was designed. Each hepatitis case was 14 matched with controls by age, gender, treatment history, disease severity and drug dosage. The tSNPs were selected by using Haploview 4.2 based on the HapMap database of Han Chinese in Beijing, and detected by using TaqMan allelic discrimination technology.

**Results:**

Eighty-nine anti-TB drug-induced hepatitis cases and 356 controls were included in this study. 6 tSNPs (rs2031920, rs2070672, rs915908, rs8192775, rs2515641, rs2515644) were genotyped and minor allele frequencies of these tSNPs were 21.9%, 23.0%, 19.1%, 23.6%, 20.8% and 44.4% in the cases and 20.9%, 22.7%, 18.9%, 23.2%, 18.2% and 43.2% in the controls, respectively. No significant difference was observed in genotypes or allele frequencies of the 6 tSNPs between case group and control group, and neither of haplotypes in block 1 nor in block 2 was significantly associated with the development of hepatitis.

**Conclusion:**

Based on the Chinese anti-TB treatment cohort, we did not find a statistically significant association between genetic polymorphisms of *CYP2E1* and the risk of anti-TB drug-induced hepatitis. None of the haplotypes showed a significant association with the development of hepatitis in Chinese TB population.

## Introduction

Tuberculosis (TB) remains a major global health problem with estimated 8.8 million incident cases and 1.45 million deaths globally in 2010, most of these occurring in developing countries [1]. China has the world’s second largest tuberculosis epidemic, behind only India [1]. The directly observed treatment, short course (DOTS) strategy is a major plank of the World Health Organization (WHO) global plans to stop TB. Guidelines recommend a drug combination regimen, including isoniazid (INH), rifampin (RIF), ethambutol (EMB), pyrazinamide (PZA) and streptomycin (SM) as the first-line agents [2], [3]. These drugs have ability to control and kill *Mycobacterium tuberculosis* effectively, but may cause various adverse effects, including liver injury, skin reactions, and gastrointestinal or neurological disorders [4], [5]. Anti-TB drug-induced hepatitis is one of the serious adverse drug reactions, which often impedes scheduled treatment and cure [6]. Anti-TB drug-induced hepatitis encompasses a wide spectrum of liver injury, ranging from asymptomatic minimal elevation of liver enzymes to acute liver failure, and often leads to death or liver transplantation [7].

Though the pathogenic mechanism of anti-TB drug-induced hepatitis is still largely obscure [8], it is suggested that reactive metabolites, rather than direct toxicities of the parent drugs, are responsible for most of idiosyncratic drug reactions [9]. Drug metabolizing enzymes have critical effects by both the synthesis and detoxification of reactive metabolites [10]. Among the first line anti-TB drugs, the metabolism of INH has been studied extensively, and it has been proposed that N-acetyl transferase 2 (NAT2), cytochrome P450 2E1 (CYP2E1) and glutathione S-transferases (GSTs) might play important roles in INH-induced hepatotoxicity [11]. In the liver, INH is first metabolized into acetylisoniazid via NAT2 [12], followed by hydrolysis to acetylhydrazine. Acetylhydrazine is then oxidised into hepatotoxic intermediaries, such as acetyldiazene, acetylonium ion, acetyl radical or ketene by CYP2E1. The hepatotoxins generated by NAT2 or CYP2E1 could be further detoxified by GSTM1 and GSTT1 presented in the liver [11].

In recent years, genetic polymorphisms of *NAT2*, *CYP2E1* as well as *GSTs* have gained more focus by linking patient’s genetic susceptibility to anti-TB drug-induced hepatitis. The slow acetylator genotype of NAT2 has been proposed to be a major risk factor for anti-TB drug-induced hepatitis in Asian populations, especially in the Chinese [13], [14], [15]. However, our recent research showed no significant associations were found between acetylator status, genotype, haplotype of NAT2 gene and anti-TB drug-induced hepatitis in a Chinese cohort [16]. The homozygous deletion (null genotype) of the *GSTM1* gene showed the lack of the GSTM1 activity and would increase risk of anti-TB drug-induced hepatitis [17], [18]. But for *CYP2E1*, results were inconsistent in China [14], [15], [19], [20]. And in other countries, all studies reported negative results about relationship between *CYP2E1* genetic polymorphisms and anti-TB drug-induced hepatitis [21], [22], [23], [24], [25], [26]. Moreover, most studies only evaluated the *Rsa*I/*Pst*I polymorphism in *CYP2E1* gene [14], [15], [19], [21], [22], [23]. So far, no tagging single-nucleotide polymorphisms (tSNPs) of *CYP2E1* in the risk of anti-TB drug-induced hepatitis have been reported.

In the present study, 6 tSNPs were selected to evaluate the association between these common genetic variants in *CYP2E1* and the risk of anti-TB drug-induced hepatitis in our established cohort of “Anti-tuberculosis Drugs induced Adverse Reactions in China National Tuberculosis Prevention and Control Scheme Study (ADACS) [27]” in a Chinese TB population.

## Methods and Design

### Patients

Anti-TB treatment patients were recruited from ADACS cohort. In brief, 4488 newly diagnosed patients with sputum smear positive pulmonary TB were recruited from four provinces (Zhejiang, Guangxi, Chongqing, Jilin) in China between October 2007 and June 2008. Diagnosis of pulmonary TB was based on chest radiographs and the isolation of *Mycobacterium tuberculosis* from a sputum smear. Before anti-tuberculosis treatment, serum hepatitis B virus surface antigen (HBsAg), serum alanine transaminase (ALT), aspartate aminotransaminase (AST), direct and total bilirubin levels, renal function, blood and urine routine test were measured. All patients took INH 600 mg, RIF 600 mg (or 450 mg if body weight was <50 kg), PZA 2000 mg and EMB 1250 mg every other day for the first 2 months (re-treatment patients were injected with SM 750 mg each time simultaneously). PZA and EMB were then discontinued for primary patients, while INH and RIF were continued for another 4 months. PZA and SM were discontinued for re-treatment patients, while INH, RIF and EMB continued. Doses did not vary with ages and sex. All patients were monitored for 6∼9 months according to the treatment episode, and liver function tests were conducted again within two months after anti-TB treatment beginning or whenever patients had symptoms of suspected hepatitis (such as anorexia, nausea, vomiting, malaise, tea-colored urine) [27]. A total of 4304 patients finished the follow-up. This study was approved by the Ethics Committee of Center for Tuberculosis Control and Prevention of China and Health Science Center of Peking University. Written informed consent was obtained from every participant or surrogate before enrolment.

Anti-TB drug-induced hepatitis was defined as: 1) an increase over two-times of the upper limit of normal (ULN) in ALT or a combined increase in AST and total bilirubin, provided one of them was more than two-times over ULN [28]; 2) causality assessment result was certain, probably or possible basing on the WHO Uppsala Monitoring Center system [29]. All suspected hepatitis patients were strictly reviewed and assessed by experts from Chinese State Food and Drug Administration.

In the present study, patients with any of the following features were excluded: 1) abnormal serum ALT, AST or total bilirubin levels before anti-TB treatment; 2) positive serum HBsAg; 3) alcoholic liver disease or habitual alcohol drinking; 4) concomitant use of hepatotoxic drugs; 5) a history of chronic liver disease or systemic diseases that may cause liver dysfunction. Among the remained patients, those in accord with the criteria of anti-TB drug-induced hepatitis were assigned into the case group, and controls were selected from those with sustained normal liver function through the whole therapy. For each case, four matched controls were identified with the same time, place, age (within 5 years old), sex, treatment history, disease severity and drug dosage.

### tSNPs Selection and Genotyping

All eligible SNPs in CYP2E1 gene including 2kb upstream and downstream of *CYP2E1* were downloaded from the Chinese Han population (CHB) database of HapMap (http://www.hapmap.org/). Then, tSNPs were selected using Haploview 4.2 software, meeting the following criteria: 1) minor allele frequency ≥0.10; 2) r^2^ of pairwise linkage disequilibrium ≥0.8. As a result, 6 tSNPs (rs2031920, rs2070672, rs915908, rs8192775, rs2515641, rs2515644) were chosen for genotyping (Table 1), which was performed by using TaqMan allelic discrimination technology on the ABI 7900 Real-Time PCR System (Applied Biosystems, Foster City, CA) [30]. The primers and probes for each tSNP were designed by Nanjing Steed BioTechnologies Co., Ltd (Table S1). Genotyping was performed by blinding the case or control status, and with a positive control of a DNA sample with a known heterozygous genotype in each test. More than 10% of the samples were repeated using the same assay and the results were 100% concordant. The overall call rate of genotyping was >98%.

**Table 1:** Information on six genotyped tSNPs of the CYP2E1 gene.

SNP No.	NCBI reference SNP No.	Chromosome Position†	Location	Base Change	MAF‡	HWE *P*-value*
1	rs2031920	135339845	5' near gene	C>T	0.268	0.440
2	rs2070672	135340548	5' near gene	A>G	0.226	0.956
3	rs915908	135346959	Intron 5	G>A	0.159	<0.001
4	rs8192775	135348026	Intron 6	G>A	0.28	0.400
5	rs2515641	135351362	exon 8	C>T	0.191	0.647
6	rs2515644	135353079	3' near gene	C>A	0.494	0.534

† SNP position in NCBI dbSNP (http://www.ncbi.nlm.nih.gov/projects/SNP).

‡ Minor allele frequency (MAF) for Han Chinese in Beijing in the HapMap database (http://www.hapmap.org).

* Hardy–Weinberg equilibrium (HWE) *P*-value in the control group.

### Statistical Analysis

Data were double entered in a database built by EpiData 3.1 (Denmark) and discrepancies were checked against the raw data. Continuous variables were described as mean ± standard deviation (SD) or median (IQR, inter-quartile range) and differences between groups were analyzed by using two-factor analysis of variance test or non-parametric test. Categorized variables were described as percentage and analyzed by using the Chi-square test. Hardy-Weinberg equilibrium was estimated using the χ^2^ goodness of fit test. Haplotype blocks were selected with Haploview 4.2 software by considering linkage disequilibrium (LD) blocks. The estimated frequency of polymorphic loci was calculated using PHASE 2.1 software. Odds ratios (OR) and 95% confidence intervals (95% CI) were calculated using conditional logistic regression model. The statistical analyses were performed with SPSS for Windows (version 13.0, SPSS Inc.). A two-tailed P-value less than 0.05 was considered statistically significant.

## Results

### Characteristics of Patients with and without Hepatitis

A total of 445 anti-TB treatment patients consisting of 89 anti-TB drug-induced hepatitis and 356 controls finished followed up were selected from ADACS cohort. All patients with HBsAg(+), regular alcohol intake, liver diseases, concomitant use of hepatotoxic drugs, and abnormal liver function before anti-TB treatment were excluded. Among 89 cases, 79 patients had over 2 times increased ALT level and 10 patients had a combined increase in AST and total bilirubin provided one of them is above 2 times of ULN. Sixty nine (77.5%) out of 89 patients with hepatitis had obvious clinical symptoms. The median time between the initiation of anti-TB treatment and the detection of hepatitis was 35 days. The further causality assessment revealed that 10 cases (11.2%) were identified as certain, 62 (69.7%) as probable and 17 (19.1%) as possible. Due to 1∶4 individual matching procedure, there was no statistical difference in the distribution of both age, weight and body mass index between cases and controls (Table 2). Before anti-TB treatment, all cases and controls had normal values of liver biochemical parameters and there was no significant difference between two groups (P>0.05). During anti-TB treatment, the peak AST, ALT, and total bilirubin levels in the cases were significantly higher compared to the values of the controls (P<0.0001).

**Table 2:** Characteristics of patients with and without anti-TB drug-induced hepatitis.

	Patients with hepatitis N = 89	Patients without hepatitis N = 356	*P*
Sex (male/female)	65/24	260/96	
treatment history(primary/re-treatment)	78/11	312/44	
Age (years)†	43.7±16.4(20.0–80.0)	43.6±16.4(17.0–84.0)	0.742§
Weight (kg)†	53.2±8.0(38.0–80.0)	53.5±7.4(31.0–84.0)	0.748§
BMI (Kg/m^2^)†	19.5±2.3(14.0–27.7)	19.4±2.3(13.5–27.0)	0.959§
Baseline value*			
ALT (U/L)‡	16.9(10.8–26.3)	16.0(10.4–22.0)	0.283¶
AST (U/L)‡	24.8(17.2–32.6)	21.4(15.3–27.0)	0.087¶
Total bilirubin (umol/L)‡	9.5(7.5–13.7)	9.7(7.4–12.5)	1.000¶
During treatment(Peak value)*			
ALT (U/L)‡	121.0(88.2–183.6)	17.0(11.6–23.2)	<0.0001¶
AST (U/L)‡	95.1(60.6–174.7)	23.7(16.7–29.0)	<0.0001¶
total bilirubin (umol/L)‡	14.3(11.4–18.0)	9.7(6.8–13.7)	<0.0001¶

* Normal intervals: ALT<40U/L, AST<40U/L, Total bilirubin<19 umol/L.

BMI, body mass index; ALT, alanine aminotransferase; AST, aspartate aminotransferase.

† Values are presented as mean±standard deviations (range).

‡ Values are presented as median (inter-quartile range).

§ two-factor analysis of variance test.

¶ Median test.

### *CYP2E1* Polymorphism in Patients with and without Hepatitis

Five of six tSNPs we genotyped were in Hardy-Weinberg equilibrium [rs2031920 (χ^2^ = 0.596, P = 0.440), rs2070672 (χ^2^ = 0.003, P = 0.956), rs8192775 (χ^2^ = 0.708, P = 0.400), rs2515641 (χ^2^ = 0.209, P = 0.647) and rs2515644 (χ^2^ = 0.387, P = 0.534)] except for rs915908 (χ^2^ = 65.242, P<0.001). The genotype analysis showed that the minor allele frequencies of rs2031920, rs2070672, rs915908, rs8192775, rs2515641 and rs2515644 were 21.9%, 23.0%, 19.1%, 23.6%, 20.8% and 44.4% in the cases and 20.9%, 22.7%, 18.9%, 23.2%, 18.2% and 43.2% in the controls, respectively. No significant difference was observed in genotypes or in allele frequencies of the six tSNPs between the case and the control group (Table 3). There was no statistically significant association between rs2031920 CC genotype (c1/c1) and anti-TB drug-induced hepatitis (OR = 0.99, 95% CI: 0.62–1.59) compared with CT or TT genotypes (c1/c2 or c2/c2).

**Table 3:** Distribution of genotypes in patients with and without anti-TB drug-induced hepatitis.

tSNPs	Patients with hepatitis		Patients without hepatitis		OR(95% CI)^a^	*P*	Model		
	N	%	N	%			model^b^	OR(95% CI)^a^	*P*
rs2031920(C>T)									
CC	56	62.9	225	63.2	1		Dom	1.012(0.630–1.624)	0.961
CT	27	30.3	113	31.7	0.957(0.577–1.587)	0.865	Rec	1.373(0.518–3.640)	0.524
TT	6	6.8	18	5.1	1.356(0.506–3.632)	0.544	Add	1.055(0.720–1.548)	0.782
rs2070672(A>G)									
AA	51	57.3	210	59.7	1		Dom	1.123(0.693–1.820)	0.638
AG	35	39.3	124	35.2	1.201(0.726–1.985)	0.475	Rec	0.641(0.179–2.294)	0.494
GG	3	3.4	18	5.1	0.676(0.187–2.446)	0.551	Add	1.030(0.690–1.538)	0.886
rs915908(G>A)									
GG	65	73.0	256	72.3	1		Dom	0.958(0.568–1.618)	0.873
AG	14	15.7	62	17.5	0.881(0.466–1.664)	0.696	Rec	1.126(0.534–2.374)	0.755
AA	10	11.2	36	10.2	1.098(0.515–2.339)	0.809	Add	1.006(0.709–1.429)	0.971
rs8192775(G>A)									
GG	51	57.3	212	59.7	1		Dom	1.107(0.695–1.762)	0.668
AG	34	38.2	121	34.1	1.180(0.724–1.922)	0.506	Rec	0.715(0.240–2.128)	0.546
AA	4	4.5	22	6.2	0.752(0.250–2.263)	0.612	Add	1.022(0.702–1.488)	0.908
rs2515641(C>T)									
CC	56	62.9	239	67.3	1		Dom	1.208(0.751–1.944)	0.436
CT	29	32.6	103	29.0	1.195(0.728–1.963)	0.481	Rec	1.239(0.396–3.874)	0.712
TT	4	4.5	13	3.7	1.310(0.414–4.143)	0.645	Add	1.174(0.787–1.750)	0.432
rs2515644(C>A)									
CC	26	29.2	117	33.1	1		Dom	1.182(0.719–1.944)	0.510
AC	47	52.8	168	47.5	1.240(0.737–2.088)	0.418	Rec	0.897(0.487–1.651)	0.726
AA	16	18.0	69	19.5	1.024(0.511–2.051)	0.946	Add	1.043(0.751–1.449)	0.801

a conditional logistic regression model analysis.

b Dom, dominant model; Rec, recessive model; Add, additive mode.

### Haplotype Analysis of *CYP2E1* in Patients with and without Hepatitis

Reconstructed linkage disequilibrium (LD) plot by using Haploview 4.2 software among the *CYP2E1* gene 6 tSNPs in 356 control subjects was shown in Figure S1. By considering both D’ and r^2^, we analyzed two haplotypes as presented in the Table 4. In block 1, Strong LD between rs2031920 and rs2070672 was observed (D’ = 1, r^2^ = 0.087). And in block 2, Strong LD between rs8192775, rs2515641 and rs2515644 was also observed (D’>0.97, r^2^>0.067). But neither of haplotypes in block 1 nor in block 2 was significantly associated with the development of hepatitis.

**Table 4:** Haplotype frequencies and the risks of anti-TB drug-induced hepatitis.

Haplotypes	Patients with hepatitis(%)	Patients without hepatitis(%)	OR(95% CI)^a^	*P*
rs2031920-rs2070672				
C-A	55.1	56.6	1	
C-G	23.0	22.4	1.054(0.700–1.587)	0.803
T-A	21.9	21.0	1.075(0.711–1.627)	0.730
rs8192775-rs2515641-rs2515644				
G-C-C	54.5	55.8	1	
A-C-A	21.9	22.5	0.992(0.657–1.499)	0.971
G-T-A	18.0	18.1	1.006(0.644–1.572)	0.977
G-C-A	2.8	2.7	1.070(0.388–2.948)	0.896
A-C-C	0.0	0.8	–	0.983
G-T-C	1.1	0.1	7.697(0.693–85.541)	0.097
A-T-A	1.7	0.0	–	0.991

a conditional logistic regression model analysis.

## Discussion

The CYP450 proteins constitute a superfamily of hemeproteins which play a central role in the oxidative metabolism and biotransformation of a variety of endogenous and exogenous compounds [31]. CYP2E1, a key member of the P450 superfamily, is one of the most important phase I metabolic enzymes and participates in the metabolism of many drugs, including anti-TB drugs. CYP2E1 can oxidate monoacetyl hydrazine and acetyl hydrazine produced from INH to generate hepatotoxins such as acetyldiazene, acetylonium ion, acetyl radical or ketene [32]. RIF is a potent inducer of CYP2E1, and it can increase the activity of this enzyme and thus regulate the production of hepatotoxic agents [11]. So higher activity of CYP2E1 may increase the synthesis of hepatotoxins and lead to hepatotoxicity.

Polymorphisms of the genes encoding CYP2E1 may influence the activity of enzyme as well as the susceptibility to anti-TB drug-induced hepatitis [19]. Among several *CYP2E1* genetic polymorphisms, only the *Rsa*I/*Pst*I (rs2031920/rs3813867) and *Dra*I (rs6413432) polymorphism have been evaluated in association with anti-TB drug-induced hepatitis [11]. The *Rsa*I and *Pst*I restriction sites are in the transcription-regulation region of *CYP2E1* and in complete linkage disequilibrium, which has been linked with gene expression [33]. Previous investigators demonstrated that subjects with *CYP2E1 Rsa*I c1/c1 genotype had higher CYP2E1 activity than those with *Rsa*I c1/c2 or c2/c2 genotype, under the inhibitory effect of isoniazid. Therefore, subjects with *CYP2E1* c1/c1 may generate more hepatotoxins and increase their risk for hepatotoxicity in Taiwanese (OR = 2.52, P = 0.009) [19]. Wang also reported that the *CYP2E1 Rsa*I c1/c1 genotype was an independent risk factor for hepatotoxicity in China (OR = 1.979, 95% CI:1.106–3.891, P = 0.023) [14]. But the results of other studies conducted in China [15], [20], India [34], Canada [23], Japan [26], Korea [21], [22], and Brazil [25] all showed no significant association between *CYP2E1 Rsa*I c1/c1 genotype and hepatitis. The polymorphism detected by *Dra*I digestion is located in intron 6, and no functional significance of this polymorphism currently is known [19]. For susceptibility to the hepatic tumor or liver disease, *Dra*I polymorphism adds no more information than *CYP2E1 Rsa*I genotypes [19], [35]. So far, only two studies reported the relationship between *CYP2E1 Dra*I polymorphism and anti-TB drug-induced hepatitis, with inconsistent findings [24], [34].

In the present study, 6 tSNPs were selected using Haploview 4.2 based on the HapMap database of Han Chinese in Beijing and evaluated the association between these genetic variants in *CYP2E1* and the risk of anti-TB drug-induced hepatitis. As we know, this is the first study to explore association between tSNPs of *CYP2E1* and anti-TB drug-induced hepatitis in Chinese TB patients. But we did not find a statistically significant association between genetic polymorphisms of *CYP2E1* and the risk of anti-TB drug-induced hepatitis. None of the haplotypes showed a significant association with the development of hepatitis. Similar to our report, another case-control study in Korea also failed to find any association of tSNPs in *CYP2E1* with anti-TB drug-induced hepatitis in Korean population [22]. In that study, three tSNPs (rs2031920, rs2070672, rs2070673) were selected, and two tSNPs (rs2031920, rs2070672) were same as our study. In addition, One of tSNPs was *Rsa*I polymorphism (rs2031920), and our results showed there was no statistically significant association between *CTY2E1 Rsa*I c1/c1 genotype and anti-TB drug-induced hepatitis (OR = 0.99, 95% CI:0.62–1.59) compared with *CYP2E1 Rsa*I c1/c2 or c2/c2 genotypes. It was similar to two other recently published studies on Chinese subjects which showed non-significant association too [15], [20].

Earlier reports showed polymorphisms of *CYP2E1* may increase the risk of drug-induced hepatitis [14], [19]. But our study could not validate these results. This discrepancy could be attributed to several reasons. First, as the pathogenic mechanism of anti-TB drug-induced hepatitis is poorly understood, most studies were based on INH metabolic pathway [11]. But INH, RIF and PZA are all hepatotoxic drugs [36] and different anti-TB drugs may have different pathogenic mechanisms [37].The patients in different studies were treated with different therapeutic regimens (DOTS or non-DOTS) or dose usage. For example, patients in our study were treated with the same standard anti-TB regimens and took drugs every other day, which was different from other reports [15], [19], [21], [22], [24]. In our study, there were 78.6% primary treatment patients and 21.4% re-treatment ones; but the treatment histories were not described in other studies [14], [19]. Second, polymorphisms of the genes encoding *CYP2E1* may influence the activity of enzyme, but efficiency change caused by a single nucleotide mutation might not be enough to alter overall promoter activity [38]. Many other factors may influence gene expression transcription and translation of *CYP2E1*, except for SNP. Gene methylation may influence the expression of CYP [39]. Vieira et al [40] reported an association between *CYP2E1* transcripts and decreased methylation of CpG residues in intron 1 of the *CYP2E1* gene that occurred during the late neonatal period. In addition microRNA (miRNA) regulation of CYP has been described [39]. Human *CYP2E1* expression is regulated by miR-378, mainly via translational repression [41]. Third, Different studied were conducted with different populations, study designs, methods of defining liver injury and standards of choosing cases and controls. Our study was designed as a case-control study nested in an anti-TB population-based cohort while others were hospital-based [14], [15], [19]. In the previous hospital-based studies, participants represented more complex and severe patients. For example, the median peak level of ALT and AST which were described in the study performed by Huang *et al* were 235 U/L and 233 U/L, respectively [19], whereas the median peak level of ALT and AST were 121.0 U/L and 95.1 U/L in our study. For the maximum value of ALT and AST, they were 2090 U/L and 2260 U/L among patients with anti-TB drug-induced hepatitis in Huang’s study, which were higher than those in our study (ALT: 691.2U/L and AST: 467.5 U/L).

One of the strengths in our study was a case-control study nested in a cohort. Due to the large sample size of the anti-TB treatment cohort, we were able to perform 1∶4 matching with aims to increase efficiency and control potential confounders. Each case was reviewed and assessed by experts strictly to define anti-TB drug-induced hepatitis. Therefore, misclassification of diagnosis was rare in our study. One potential limitation is the small sample size in our study. Because some patients with HBsAg (+), alcohol drinking, liver diseases, and so on were excluded from this study. But this problem existed in other similar studies, which had relatively small sample sizes ranging from 8 [34] to 104 [14] patients in case group. Another limitation is that we did not collect patients’ information of prior hepatitis C infection history which may be a risk factor for hepatotoxicity.

### Conclusions

This is the first exploration of an association between tSNPs of *CYP2E1* and anti-TB drug-induced hepatitis in Chinese TB patients. Based on a case-control study nested in the ADACS cohort, we did not find a statistically significant association between genetic polymorphisms of *CYP2E1* and the risk of anti-TB drug-induced hepatitis. None of the haplotypes showed a significant association with the development of hepatitis in the Chinese TB population.

## Supporting Information

Figure S1 **Linkage disequilibrium plot for selected SNPs of** ***CYP2E1*** **gene.** The plot is generated by Haploview software. D’ values are shown on the squares. The colors of the squares represent r^2^ values, with dark being r^2^ = 1, and white being r^2^ = 0.
(TIF)

Table S1 **Information of primers and probes.**
(DOCX)

## References

[1] World Health Organization (2011) Global tuberculosis control: WHO report 2011. Geneva: World Health Organization. 1–11.

[2] Blumberg HM, Burman WJ, Chaisson RE, Daley CL, Etkind SC, et al (2003) American Thoracic Society/Centers for Disease Control and Prevention/Infectious Diseases Society of America: treatment of tuberculosis. Am J Respir Crit Care Med 167: 603–662. 12588714 10.1164/rccm.167.4.603

[3] Joint Tuberculosis Committee of the British Thoracic Society (1998) Chemotherapy and management of tuberculosis in the United Kingdom: recommendations 1998. Thorax 53: 536–548. 9797751 PMC1745276

[4] Burman WJ, Reves RR (2001) Hepatotoxicity from rifampin plus pyrazinamide: lessons for policymakers and messages for care providers. Am J Respir Crit Care Med 164: 1112–1113. 11673194 10.1164/ajrccm.164.7.2109052

[5] Yee D, Valiquette C, Pelletier M, Parisien I, Rocher I, et al (2003) Incidence of serious side effects from first-line antituberculosis drugs among patients treated for active tuberculosis. Am J Respir Crit Care Med 167: 1472–1477. 12569078 10.1164/rccm.200206-626OC

[6] Saukkonen JJ, Cohn DL, Jasmer RM, Schenker S, Jereb JA, et al (2006) An official ATS statement: hepatotoxicity of antituberculosis therapy. Am J Respir Crit Care Med 174: 935–952. 17021358 10.1164/rccm.200510-1666ST

[7] Devarbhavi H (2011) Antituberculous drug-induced liver injury: current perspective. Trop Gastroenterol 32: 167–174. 22332331

[8] Yew WW, Leung CC (2006) Antituberculosis drugs and hepatotoxicity. Respirology 11: 699–707. 17052297 10.1111/j.1440-1843.2006.00941.x

[9] Knowles SR, Uetrecht J, Shear NH (2000) Idiosyncratic drug reactions: the reactive metabolite syndromes. Lancet 356: 1587–1591. 11075787 10.1016/S0140-6736(00)03137-8

[10] Naisbitt DJ, Williams DP, Pirmohamed M, Kitteringham NR, Park BK (2001) Reactive metabolites and their role in drug reactions. Curr Opin Allergy Clin Immunol 1: 317–325. 11964707 10.1097/01.all.0000011033.64625.5a

[11] Roy PD, Majumder M, Roy B (2008) Pharmacogenomics of anti-TB drugs-related hepatotoxicity. Pharmacogenomics 9: 311–321. 18303967 10.2217/14622416.9.3.311

[12] Mitchell JR, Zimmerman HJ, Ishak KG, Thorgeirsson UP, Timbrell JA, et al (1976) Isoniazid liver injury: clinical spectrum, pathology, and probable pathogenesis. Ann Intern Med 84: 181–192. 766682 10.7326/0003-4819-84-2-181

[13] Huang YS, Chern HD, Su WJ, Wu JC, Lai SL, et al (2002) Polymorphism of the N-acetyltransferase 2 gene as a susceptibility risk factor for antituberculosis drug-induced hepatitis. Hepatology 35: 883–889. 11915035 10.1053/jhep.2002.32102

[14] Wang T, Yu HT, Wang W, Pan YY, He LX, et al (2010) Genetic polymorphisms of cytochrome P450 and glutathione S-transferase associated with antituberculosis drug-induced hepatotoxicity in Chinese tuberculosis patients. J Int Med Res 38: 977–986. 20819434 10.1177/147323001003800324

[15] Lee SW, Chung LS, Huang HH, Chuang TY, Liou YH, et al (2010) NAT2 and CYP2E1 polymorphisms and susceptibility to first-line anti-tuberculosis drug-induced hepatitis. Int J Tuberc Lung Dis 14: 622–626. 20392357

[16] Lv X, Tang S, Xia Y, Zhang Y, Wu S, et al (2012) NAT2 genetic polymorphisms and anti-tuberculosis drug-induced hepatotoxicity in Chinese community population. Ann Hepatol 11: 700–707. 22947533

[17] Huang YS, Su WJ, Huang YH, Chen CY, Chang FY, et al (2007) Genetic polymorphisms of manganese superoxide dismutase, NAD(P)H:quinone oxidoreductase, glutathione S-transferase M1 and T1, and the susceptibility to drug-induced liver injury. J Hepatol 47: 128–134. 17400324 10.1016/j.jhep.2007.02.009

[18] Guo M, Sun YH, Li SM, Wang D, Liu Q, et al (2009) The effect of GST M1 and GST T1 gene mutations on anti-tuberculous drug induced hepatic injury. Chinese journal of tuberculosis and respiratory diseases 32: 266–269. 19576039

[19] Huang YS, Chern HD, Su WJ, Wu JC, Chang SC, et al (2003) Cytochrome P450 2E1 genotype and the susceptibility to antituberculosis drug-induced hepatitis. Hepatology 37: 924–930. 12668988 10.1053/jhep.2003.50144

[20] An HR, Wu UQ, Wang ZY, Zhang JX, Liang Y (2012) NAT 2 and CYP2E1 polymorphisms associated with antituberculosis drug-induced hepatotoxicity in Chinese patients. Clin Exp Pharmacol Physiol 39: 535–543. 22506592 10.1111/j.1440-1681.2012.05713.x

[21] Cho HJ, Koh WJ, Ryu YJ, Ki CS, Nam MH, et al (2007) Genetic polymorphisms of NAT2 and CYP2E1 associated with antituberculosis drug-induced hepatotoxicity in Korean patients with pulmonary tuberculosis. Tuberculosis (Edinb) 87: 551–556. 17950035 10.1016/j.tube.2007.05.012

[22] Kim SH, Bahn JW, Kim YK, Chang YS, Shin ES, et al (2009) Genetic polymorphisms of drug-metabolizing enzymes and anti-TB drug-induced hepatitis. Pharmacogenomics 10: 1767–1779. 19891553 10.2217/pgs.09.100

[23] Yamada S, Tang M, Richardson K, Halaschek-Wiener J, Chan M, et al (2009) Genetic variations of NAT2 and CYP2E1 and isoniazid hepatotoxicity in a diverse population. Pharmacogenomics 10: 1433–1445. 19761367 10.2217/pgs.09.66

[24] Bose PD, Sarma MP, Medhi S, Das BC, Husain SA, et al (2011) Role of polymorphic N-acetyl transferase2 and cytochrome P4502E1 gene in antituberculosis treatment-induced hepatitis. J Gastroenterol Hepatol 26: 312–318. 21261721 10.1111/j.1440-1746.2010.06355.x

[25] Teixeira RL, Morato RG, Cabello PH, Muniz LM, Moreira Ada S, et al (2011) Genetic polymorphisms of NAT2, CYP2E1 and GST enzymes and the occurrence of antituberculosis drug-induced hepatitis in Brazilian TB patients. Mem Inst Oswaldo Cruz 106: 716–724. 22012226 10.1590/s0074-02762011000600011

[26] Sotsuka T, Sasaki Y, Hirai S, Yamagishi F, Ueno K (2011) Association of isoniazid-metabolizing enzyme genotypes and isoniazid-induced hepatotoxicity in tuberculosis patients. In Vivo 25: 803–812. 21753138

[27] Xia YY, Hu DY, Liu FY, Wang XM, Yuan YL, et al (2010) Design of the anti-tuberculosis drugs induced adverse reactions in China National Tuberculosis Prevention and Control Scheme Study (ADACS). BMC Public Health 10: 267–276. 20492672 10.1186/1471-2458-10-267 PMC2893093

[28] Benichou C (1990) Criteria of drug-induced liver disorders. Report of an international consensus meeting. J Hepatol 11: 272–276. 2254635 10.1016/0168-8278(90)90124-a

[29] World Health Organization (2005) The use of the WHO-UMC system for standardized case causality assessment. Uppsala: The Uppsala Monitoring Centre.

[30] Teuber M, Wenz MH, Schreiber S, Franke A (2009) GMFilter and SXTestPlate: software tools for improving the SNPlex genotyping system. BMC Bioinformatics 10: 81. 19267942 10.1186/1471-2105-10-81 PMC2661053

[31] Jana S, Paliwal J (2007) Molecular mechanisms of cytochrome p450 induction: potential for drug-drug interactions. Curr Protein Pept Sci 8: 619–628. 18220847 10.2174/138920307783018668

[32] Nelson SD, Mitchell JR, Timbrell JA, Snodgrass WR, Corcoran GB, 3rd (1976) Isoniazid and iproniazid: activation of metabolites to toxic intermediates in man and rat. Science 193: 901–903. 7838 10.1126/science.7838

[33] Hayashi S, Watanabe J, Kawajiri K (1991) Genetic polymorphisms in the 5'-flanking region change transcriptional regulation of the human cytochrome P450IIE1 gene. J Biochem 110: 559–565. 1778977 10.1093/oxfordjournals.jbchem.a123619

[34] Roy B, Ghosh SK, Sutradhar D, Sikdar N, Mazumder S, et al (2006) Predisposition of antituberculosis drug induced hepatotoxicity by cytochrome P450 2E1 genotype and haplotype in pediatric patients. J Gastroenterol Hepatol 21: 784–786. 16677176 10.1111/j.1440-1746.2006.04197.x

[35] Tsutsumi M, Takada A, Wang JS (1994) Genetic polymorphisms of cytochrome P4502E1 related to the development of alcoholic liver disease. Gastroenterology 107: 1430–1435. 7926507 10.1016/0016-5085(94)90546-0

[36] Tostmann A, Boeree MJ, Aarnoutse RE, de Lange WC, van der Ven AJ, et al (2008) Antituberculosis drug-induced hepatotoxicity: concise up-to-date review. J Gastroenterol Hepatol 23: 192–202. 17995946 10.1111/j.1440-1746.2007.05207.x

[37] De Rosa HJ, Baldan HM, Brunetti IL, Ximenes VF, Machado RG (2007) The effect of pyrazinamide and rifampicin on isoniazid metabolism in rats. Biopharm Drug Dispos 28: 291–296. 17571294 10.1002/bdd.557

[38] Huang X, Chen L, Song W, Niu J, Han X, et al (2012) Systematic Functional Characterization of Cytochrome P450 2E1 Promoter Variants in the Chinese Han Population. PLoS One 7: e40883. 22815852 10.1371/journal.pone.0040883 PMC3398937

[39] Ingelman-Sundberg M, Sim SC, Gomez A, Rodriguez-Antona C (2007) Influence of cytochrome P450 polymorphisms on drug therapies: pharmacogenetic, pharmacoepigenetic and clinical aspects. Pharmacol Ther 116: 496–526. 18001838 10.1016/j.pharmthera.2007.09.004

[40] Vieira I, Sonnier M, Cresteil T (1996) Developmental expression of CYP2E1 in the human liver. Hypermethylation control of gene expression during the neonatal period. Eur J Biochem 238: 476–483. 8681961 10.1111/j.1432-1033.1996.0476z.x

[41] Mohri T, Nakajima M, Fukami T, Takamiya M, Aoki Y, et al (2010) Human CYP2E1 is regulated by miR-378. Biochem Pharmacol 79: 1045–1052. 19945440 10.1016/j.bcp.2009.11.015

