# Supplementary figures and images for: Cytochrome P450 2E1 Gene Polymorphisms/Haplotypes and Anti-Tuberculosis Drug-Induced Hepatitis in a Chinese Cohort
Source: PLoS One. 2013 Feb 27;8(2):e57526. doi: 10.1371/journal.pone.0057526 (PMC3583841; doi:10.1371/journal.pone.0057526)

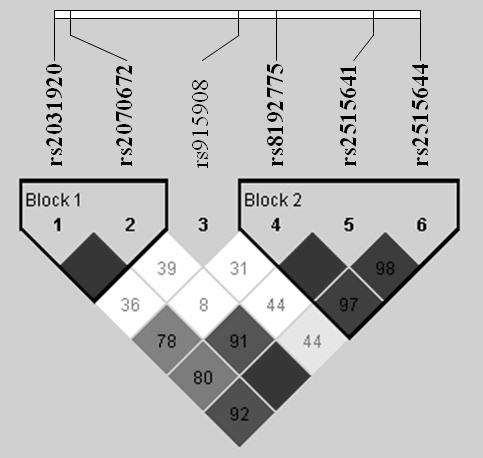

Supplement: Figure S1 — Linkage disequilibrium plot for selected SNPs of CYP2E1 gene. The plot is generated by Haploview software. D’ values are shown on the squares. The colors of the squares represent r2 values, with dark being r2 = 1, and white being r2 = 0. (TIF) [file pone.0057526.s001.tif]
