# Supplementary material for: Cytochrome P450 2E1 Gene Polymorphisms/Haplotypes and Anti-Tuberculosis Drug-Induced Hepatitis in a Chinese Cohort
Source: PLoS One. 2013 Feb 27;8(2):e57526. doi: 10.1371/journal.pone.0057526 (PMC3583841; doi:10.1371/journal.pone.0057526)
Supplement: Table S1 — Information of primers and probes. (DOCX) [file pone.0057526.s002.docx]

Table S1 Information of primers and probes.

| tSNPs | Primer sequence (5’-3’) | Probe sequence |
| --- | --- | --- |
| rs2031920  C>T | F-GGCTGGATTGTAAATGACTTTTATTT  R-TTTGTGTGTGTGGTTAGAATGAAGAG | C:FAM-ATAAAAGTACAAAATTGC-MGB  T: HEX-AATATAAAAGTATAAAATTGC-MGB |
|  |  |  |
| rs2070672  A>G | F-ACATAAACCCTACTCCAAACAAATGCA  R-TTGCCAACCCATAGTTAAGAACGT | C: FAM-TGGCACTGGCTAGACA-MGB  T: HEX-TTTGGCACTGGTTAGACA-MGB |
|  |  |  |
| rs915908  G>A | F-CAGCAGACACTAGCCCTGTAG  R-CCCCTGACCTTGGTCATAGC | C: FAM-AGCTAAACGGCTCCTGC-MGB  T: HEX-AGCTAAACGGTTCCTGC-MGB |
|  |  |  |
| rs8192775  G>A | F-GGCCAAGTAGAGGTGATGTGA  R-ACTTGGACCCAACCCTGACT | C: FAM- TTTGCATGCGGGTGC-MGB  T: HEX-TGTTTGCATGTGGGTGC-MGB |
|  |  |  |
| rs2515641  C>T | F-AGCCAGAACACTTCCTGAATGAAAA  R-CACCTGTGGAAAATGGCTTGAAAT | A: FAM-CACTGTACTTAAACTTT-MGB  G: HEX-CACTGTACTTGAACTTT-MGB |
|  |  |  |
| rs2515644  C>A | F-ACATAATTTTCATGTTATTGAAAAGCTCTTCCATC  R-TCCCCACCTCAATATGATTCTGACA | C: FAM-AGGATCACACCACTTCA-MGB  A: HEX-TAGGATCACACAACTTCA-MGB |
